# Medical Lexicon; a New Dictionary of Medical Science, Containing a Concise Account of the Various Subjects and Terms; with a Vocabulary of Synonymes, &c.

**Published:** 1839-10

**Authors:** 


Art. XIX.—*Medical Lexicon; a new Dictionary of Medical Science, containing a concise account of the various Subjects and Terms; with a Vocabulary of Synonymes in different Languages, and Formulæ for various Officinal and Empirical Preparations, &c. Second Edition, with numerous modifications and additions.* By Robley Dunglison, m.d., &c.—*Philadelphia,* 1839. 8vo, pp. 821.

The first edition of this work was published in 1833, in two volumes, and has ever since been the standard authority for reference with American students. It reappears in a single volume, considerably altered, and much improved. All the biographical and bibliographical articles are omitted, while a vast number of new words are added; and the scientific and medical definitions and descriptions are modified, according to the present state of knowledge or opinions. This dictionary is on the same plan as Dr. Hooper's, being not "a mere lexicon or dictionary of terms, but affording, under each, a condensed view of its various medical relations, and thus rendering the work an epitome of the existing condition of medical science." (Preface.) Being only of about half the size of Dr. Grant's edition of Hooper, its scientific and medical articles are necessarily much less perfect; but we hardly consider this as a defect, as the knowledge of systematised *facts* must be sought elsewhere than in dictionaries. There is, appended to the work, a list of words, termed "Index to the synonymes," extending over nearly 200 pages, and containing, the author tells us, more than 20,000 vocables, which is a feature peculiar to this dictionary, but which ought to be found in all works of this class. The provoking difficulty of being unable to find the word you are looking for, so constantly experienced in consulting dictionaries, is, in a considerable degree, obviated by this arrangement. Professor Dunglison's Lexicon will be of infinite use to medical students, and we recommend it to the attention of the ardent alumni of the hundred medical schools throughout the Union.

